# Tip-Enhanced Raman Spectroscopy with High-Order Fiber Vector Beam Excitation

**DOI:** 10.3390/s18113841

**Published:** 2018-11-09

**Authors:** Fanfan Lu, Tengxiang Huang, Lei Han, Haisheng Su, Heng Wang, Min Liu, Wending Zhang, Xiang Wang, Ting Mei

**Affiliations:** 1 MOE Key Laboratory of Material Physics and Chemistry under Extraordinary Conditions and Shaanxi Key Laboratory of Optical Information Technology, School of Science, Northwestern Polytechnical University, Xi’an 710072, China; lufanfan@mail.nwpu.edu.cn (F.L.); hanlei604@mail.nwpu.edu.cn (L.H.); nwpuwh@163.com (H.W.); liumin@mail.nwpu.edu.cn (M.L.); 2 State Key Laboratory of Physical Chemistry of Solid surface, Department of Chemistry, College of Chemistry and Chemical Engineering, Xiamen University, Xiamen 361005, China; txhuang1@163.com (T.H.); suhaisheng512@163.com (H.S.); wangxiang@xmu.edu.cn (X.W.)

**Keywords:** tip-enhanced Raman spectroscopy, field enhancement, fiber vector beam

## Abstract

We investigated tip-enhanced Raman spectra excited by high-order fiber vector beams. Theoretical analysis shows that the high-order fiber vector beams have stronger longitudinal electric field components than linearly polarized light under tight focusing conditions. By introducing the high-order fiber vector beams and the linearly polarized beam from a fiber vector beam generator based on an electrically-controlled acoustically-induced fiber grating into a top-illumination tip-enhanced Raman spectroscopy (TERS) setup, the tip-enhanced Raman signal produced by the high-order fiber vector beams was 1.6 times as strong as that produced by the linearly polarized light. This result suggests a new type of efficient excitation light beams for TERS.

## 1. Introduction

In recent years, tip-enhanced Raman spectroscopy (TERS) has attracted much attention due to its high spatial resolution with nanometer scale and high detection sensitivity, even at the single molecule level [1,2,3,4,5]. In addition to its capability of recording topographic and chemical fingerprint information of surfaces simultaneously, potential applications of TERS have been widely explored in areas of the surface science [6,7], low-dimension materials [8,9,10,11], biological systems [12,13,14,15,16], molecular electronics [17,18], catalysis [19,20,21], art conservation [22], etc.

It is generally known that the polarization of the excitation light plays a crucial role in producing the tip-enhanced Raman (TER) signal, whose intensity is mainly determined by the localized surface plasmon resonance (LSPR) [23,24,25,26] and the lightning rod effect [27] at apex of the metallic tip. For an elongated metallic tip, only the electric field component parallel to the axis of the metallic tip can effectively excite the LSPR and cause the lightning rod effect. Thus, it is an effective way to obtain strong TER signals by illuminating the metallic tip with a light beam having strong longitudinal field components under condition of tight focusing. In 2004, Kawata et al. [28] introduced a quasi-radially polarized beam generated by a four-section polarizer to an inverted-illumination TERS configuration and then obtained a stronger TERS signal compared with linearly polarized light excitation, because the quasi-radially polarized beam had a stronger longitudinal component than linearly polarized light [29,30,31,32]. In 2014, Zhang et al. [33] experimentally verified the longitudinal field excited TERS enhancement using transmission-mode TERS setup, which is six times higher than that with focused linearly polarized light excitation. To date, most of these studies have focused on application of radial polarized vector beams, which are generated in free space by using the waveplates or spatial light modulators on TERS systems. In addition, the tightly focused higher-mode beam excitation for TERS is still an attractive research direction.

In this paper, we investigated TER spectra excited by high-order fiber vector beams based on an electrically-controlled tunable acoustically-induced fiber grating. Theoretical analysis shows that the high-order fiber vector beams have a stronger longitudinal electric field component than the linearly polarized light under condition of tight focusing. In the experiment, the high-order fiber vector beams and the linearly polarized beam were introduced into a top-illumination TERS configuration, and the TER spectra obtained by using the high-order fiber vector beams is stronger than that using the linearly polarized light beam. The results would be a promising reference for developing TERS techniques and suggest a new way to improve the sensitivity of TERS techniques and polarization Raman microscopy.

## 2. Theoretical Analysis

**Figure 1:**
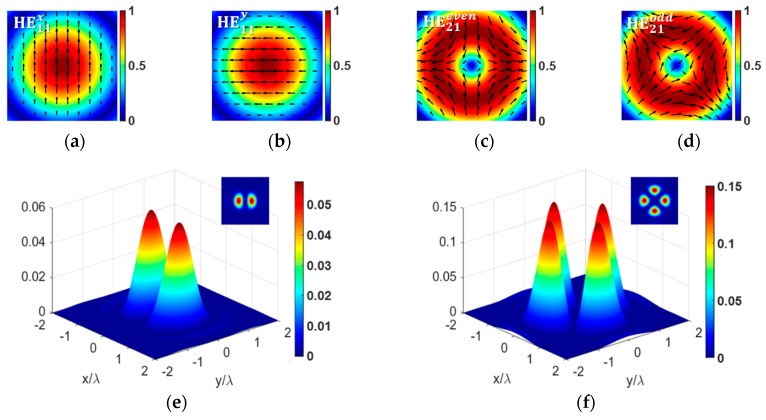
(**a**–**d**) Modal intensity distributions of ${\mathrm{HE}}_{11}^{x/y}$ and ${\mathrm{HE}}_{21}^{even/odd}$ modes with arrows denoting the polarization directions; (**e**,**f**) calculated longitudinal electric field components of ${\mathrm{HE}}_{11}^{x}$ and ${\mathrm{HE}}_{21}^{even}$ modes under condition of tight focusing.

In a few-mode fiber (FMF), the transverse electric field of a vector mode can be expressed in the weakly guiding approximation [34,35]

(1) $$E=F_{\ell m}\left(r\right)\Phi \left(\phi \right)$$

where *F_ℓm_*(*r*), (*ℓ* = 0, 1, 2…, *m* = 1, 2, 3…) is the radial distribution function of the scalar mode LP*_ℓm_*, with *ℓ* and *m* being the azimuthal and radial numbers, respectively, **Φ**(ϕ) is the field direction function, and *r* and ϕ are the radial and azimuthal coordinates, respectively. The field direction functions of the fundamental vector modes (${\mathrm{HE}}_{11}^{x/y}$) and the high-order vector modes ${\mathrm{HE}}_{21}^{even/odd}$ can be expressed as

(2) $$\left\{\begin{array}{c} {\mathrm{HE}}_{11}^{x}\\ {\mathrm{HE}}_{11}^{y} \end{array}\right\}=F_{01}\left(r\right)\left\{\begin{array}{c} \hat{x}\\ \hat{y} \end{array}\right\}$$

and

(3) $$\left\{\begin{array}{c} {\mathrm{HE}}_{21}^{even}\\ {\mathrm{HE}}_{21}^{odd} \end{array}\right\}=F_{11}\left(r\right)\left\{\begin{array}{c} \hat{x}\cos\phi -\hat{y}\sin\phi \\ \hat{x}\sin\phi +\hat{y}\cos\phi \end{array}\right\}$$

respectively, with $\hat{x}$ and $\hat{y}$ being the unit vectors. The transverse modal intensity distributions of ${\mathrm{HE}}_{11}^{x/y}$ and ${\mathrm{HE}}_{21}^{even/odd}$ modes can be calculated according to Equations (2) and (3), and are exhibited in Figure 1a–d, respectively. Figure 1a,b show the modal intensity distributions of the ${\mathrm{HE}}_{11}^{x}$ and ${\mathrm{HE}}_{11}^{y}$ modes, which are a pair of strictly degenerate vector modes with orthogonal linear polarization directions. Figure 1c,d shows the modal intensity distributions of ${\mathrm{HE}}_{21}^{even}$ and ${\mathrm{HE}}_{21}^{odd}$ modes, which are also a pair of strictly degenerate vector modes and have a *π*/4 rotation of the polarization distributions [36].

Under the tight focusing condition, longitudinal electric field components of the ${\mathrm{HE}}_{11}^{x/y}$ and ${\mathrm{HE}}_{21}^{even/odd}$ modes were calculated based on the Richards–Wolf theory [37,38]. Because ${\mathrm{HE}}_{\ell +1,1}^{even}$ and ${\mathrm{HE}}_{\ell +1,1}^{odd}$ (*ℓ* = 0, ${\mathrm{HE}}_{21}^{even/odd}$. denoted as ${\mathrm{HE}}_{11}^{x/y}$) have the same modal intensity distribution, except that there is a *π*/(*ℓ* + 1) rotation between the polarization distributions of the two degenerate modes, only the longitudinal electric field components of ${\mathrm{HE}}_{11}^{x}$ and ${\mathrm{HE}}_{\ell +1,1}^{even}$ were given under tight focusing condition with the incident wavelength λ = 633 nm, and the corresponding results are shown in Figure 1e,f, respectively. Note that the longitudinal component of ${\mathrm{HE}}_{11}^{x}$ mode has two lobes at the focal plane with zero intensity in the middle. As reported in Reference [30], this zero intensity leads to disability to excite LSPR at the metallic tip apex when the tip is located in the center of the focal region, whereas the tip-enhanced Raman signal should be better observed by locating the tip at either lobes of the focused beam. As for the ${\mathrm{HE}}_{21}^{even}$ modes, owing to the cylindrical symmetry of polarization distribution, the longitudinal component in the tightly focused field has four lobes. Although the intensity at the center is zero either, the maximum intensity of each lobe is more than twice of that of ${\mathrm{HE}}_{11}^{x}$ mode. The stronger longitudinal electric field components of ${\mathrm{HE}}_{21}^{even/odd}$ modes lead to better field enhancement than ${\mathrm{HE}}_{11}^{x/y}$ modes. Therefore, the ${\mathrm{HE}}_{21}^{even/odd}$ modes may result in stronger field enhancement than the ${\mathrm{HE}}_{11}^{x/y}$ modes.

## 3. Experimental Setup

**Figure 2:**
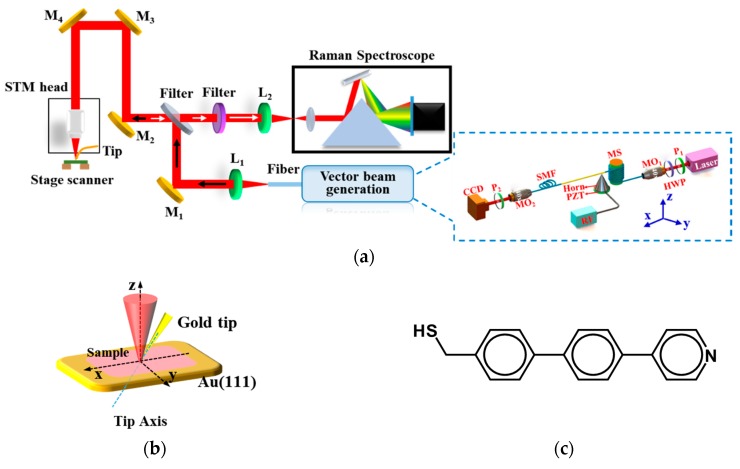
(**a**) Experiment configuration of tip-enhanced Raman spectroscopy (TERS) excited by the high-order fiber vector beams. Inset: setup for the high-order fiber vector beam generator based on electrically-controlled acoustically-induced fiber grating; (**b**) partial enlarged detail of the metallic tip and sample; (**c**) chemical structure of the 4-PBT (4-thiol-4’-(4-pyridine)biphenyl).

Figure 2a shows the experimental configuration of the TER signal excited by the high-order fiber vector beams (${\mathrm{HE}}_{21}^{even/odd}$) generated via an acoustically-induced fiber grating (AIFG). The experimental configuration of the high-order fiber vector beam generator based on an electrically-controlled tunable AIFG is shown as inset in Figure 2a. A laser with wavelength of 633 nm is used as the light source. The light is linearly polarized by a horizontal polarizer (P_1_) with the polarization orientation adjusted by a half-wave plate (HWP) to determine the launching of either ${\mathrm{HE}}_{11}^{x}$ or ${\mathrm{HE}}_{11}^{y}$ subsequently. A power of 0.8 mW was measured for the light before injecting into the few-mode fiber (FMF) through a micro-objective lens (MO_1_). Moreover, to further eliminate the effects of unwanted high-order vector modes before the AIFG, a mode tripper (MS), which was made of eight turns of FMF wound on a 4-mm diameter rod, was used to ensure a pure ${\mathrm{HE}}_{11}^{x/y}$ mode launching. When the light propagates through the mode stripper (MS), there is only the linearly polarized mode (${\mathrm{HE}}_{11}^{x}$ or ${\mathrm{HE}}_{11}^{y}$) with a power of 0.5 mW left in the fiber core. One end of the unjacketed FMF, UV epoxy was glued to the tip of the acoustic transducer, and the other was fixed on a fiber clamp. By tuning the voltage and the frequency of the radio frequency (RF) driving signal applied on the acoustic transducer, the ${\mathrm{HE}}_{11}^{x}$ (${\mathrm{HE}}_{11}^{y}$) mode was coupled to the ${\mathrm{HE}}_{21}^{even}$ (${\mathrm{HE}}_{21}^{odd}$) mode by the AIFG, when the phase-matching condition was satisfied [34,39]. The FMF output terminal was collimated using a 40 × micro-objective lens (MO_2_) and the ${\mathrm{HE}}_{21}^{even/odd}$ mode intensity patterns were recorded using a charge coupled device (CCD). Furthermore, a linear polarizer (P_2_) was inserted between the MO_2_ and the CCD to examine the modal field polarization distributions.

After the examination of polarization characteristic, the MO_2_, P_2_, and CCD were replaced by a lens (L_1_) to introduce the generated vector beam into an integration of the scanning tunneling microscopy (STM) and confocal microscopy/Raman scattering spectroscopy (NT-MDT, NTEGRA Spectra, Russia) for TER spectrum excitation. A gold tip was controlled by the device of STM to approach the Au (111) surface with adsorbed probe molecules for near-field excitation of Raman signal. The incident beam was tightly focused on the tilted metallic tip apex by a high-NA micro-objective lens (100×, NA = 0.7), as shown in Figure 2b. A piezo-stage actuator was used for rapid optical alignment between the laser spot and the gold tip apex. As the gold tip approached the vicinity of the sample surface, the Raman signal was locally enhanced and scattered to the far field. The scattered Raman signal was collected using the same micro-objective, and then coupled into a Raman spectrometer for detection.

## 4. Experimental Results and Discussion

**Figure 3:**
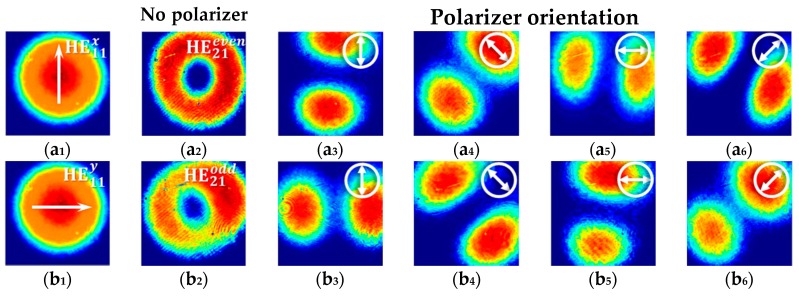
(**a_1_**,**b_1_**) Images ${\mathrm{HE}}_{11}^{x}$ of ${\mathrm{HE}}_{11}^{y}$ and modes taken by an charge coupled device (CCD) in absence of a polarizer; (**a_2_**,**b_2_**) Images of ${\mathrm{HE}}_{21}^{even}$ and ${\mathrm{HE}}_{21}^{odd}$ modes taken by a CCD in absence of a polarizer; (**a_3_**–**a_6_**,**b_3_**–**b_6_**) Images of ${\mathrm{HE}}_{21}^{even}$ and ${\mathrm{HE}}_{21}^{odd}$ modes in presence of polarizer at different polarization orientations. The image sizes are 2.5 mm × 2.5 mm.

When the RF driving signal was turned off, the fiber vector beam generator output the linearly polarized fundamental modes of ${\mathrm{HE}}_{11}^{x/y}$. Images of their intensity patterns were taken by a CCD camera as shown in Figure 3a_1_,b_1_, respectively. Selection between ${\mathrm{HE}}_{11}^{x}$ and ${\mathrm{HE}}_{11}^{y}$ was realized by rotating the HWP. In order to generate the high-order fiber vector modes of ${\mathrm{HE}}_{21}^{even/odd}$, an acoustic flexural wave was generated by the PZT being actuated by an RF driving signal with *f* = 0.8289 MHz [34], and amplified at the tip of the horn-like transducer. The output beams were projected on the CCD covered by a polarizer P_2_ to examine the mode patterns, and images of the intensity patterns at various polarizations were shown in Figure 3(a_3_–a_6_),(b_3_–b_6_), respectively.

The TER experiments were carried out with illumination of linearly polarized beams (${\mathrm{HE}}_{11}^{x}$) and high-order vector beams (${\mathrm{HE}}_{21}^{even/odd}$), respectively. The sample was prepared by adsorbing the 4-PBT (4-thiol-4′-(4-pyridine) biphenyl) on the Au (111) surface, as shown in Figure 2b. The gold tip was etched by using the electrochemical etching method [40], and the chemical structure of 4-PBT was shown in Figure 2c.

**Figure 4:**
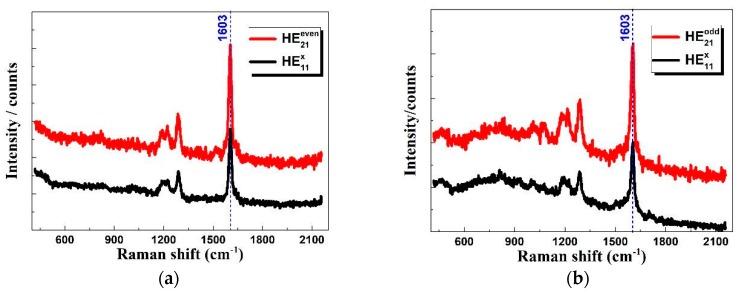
Tip-enhanced Raman spectra of 4-PBT with different excitation beams, (**a**) ${\mathrm{HE}}_{21}^{even}$ and ${\mathrm{HE}}_{11}^{x}$; (**b**) ${\mathrm{HE}}_{21}^{odd}$ and ${\mathrm{HE}}_{11}^{x}$.

Figure 4 shows the measured TER spectra of 4-PBT excited with ${\mathrm{HE}}_{11}^{x}$ and ${\mathrm{HE}}_{21}^{even/odd}$. It could be known that the Raman signal was effectively excited and enhanced with ${\mathrm{HE}}_{11}^{x}$ and ${\mathrm{HE}}_{21}^{even/odd}$ modes. Because of the far field Raman spectra of 4-PBT molecule was not visible, Figure 4 does not show its spectra when the tip retracted. Strong local enhancement of the near-field Raman signal was achieved with the gap-mode TERS [41,42]. Moreover, the Raman intensity at 1603 cm^−1^ under ${\mathrm{HE}}_{21}^{even/odd}$ illumination was 1.6 times stronger than that under ${\mathrm{HE}}_{11}^{x}$ illumination. The signal-to-noise ratios at peak 1603 cm^−1^ are calculated to be 8.45:1 and 9.34:1 under ${\mathrm{HE}}_{11}^{x}$ and ${\mathrm{HE}}_{21}^{even/odd}$ illuminations, respectively. The experimental results are consistent with the theoretical analysis, and indicate that the high-order fiber vector beams (${\mathrm{HE}}_{21}^{even/odd}$) could be used to achieve stronger Raman signal enhancement than the linearly polarized beam. Due to energy loss in the process of coupling HE_11_ mode to HE_21_ mode, the noise increases in case of higher order modes when normalize the excitation power. Compared with radial beam excitations, Raman signal enhancement was weaker, but it could be useful to polarization-controlled Raman spectroscopy due to its unique polarization property [43]. In addition, with the ${\mathrm{HE}}_{21}^{even/odd}$ modes excitation, Raman enhancement can be further boosted by optimizing the optical configuration, such as using the inverted microscope to better align the longitudinal component of the focused beam on the apex of the metallic tip for exciting the LSPR and using a micro-objective with larger NA to more tightly focus the incident beam and efficiently collect the Raman signal.

## 5. Conclusions

In summary, we experimentally demonstrated the high-order fiber vector beams can be used in TERS system. Theoretical analysis shows that the high-order fiber vector beams have stronger longitudinal electric field components than the linearly polarized light under the tight focusing condition. In the experiment, the linearly polarized beam and the high-order vector beam were introduced into a top-illumination TERS system for comparing the enhancement characteristic of TERS signal. The ${\mathrm{HE}}_{21}^{even/odd}$ modes excitation produced stronger TER signal, which was 1.6 times stronger than that produced by the ${\mathrm{HE}}_{11}^{x}$ beam, showing stronger interaction between the high-order vector beam and the metallic tip. This result will be a promising reference for the tip enhanced and polarization Raman spectroscopy.
